# Electrocardiogram Monitoring Wearable Devices and Artificial-Intelligence-Enabled Diagnostic Capabilities: A Review

**DOI:** 10.3390/s23104805

**Published:** 2023-05-16

**Authors:** Luca Neri, Matt T. Oberdier, Kirsten C. J. van Abeelen, Luca Menghini, Ethan Tumarkin, Hemantkumar Tripathi, Sujai Jaipalli, Alessandro Orro, Nazareno Paolocci, Ilaria Gallelli, Massimo Dall’Olio, Amir Beker, Richard T. Carrick, Claudio Borghi, Henry R. Halperin

**Affiliations:** 1 Department of Medicine, Division of Cardiology, Johns Hopkins University, Baltimore, MD 21218, USA; lneri1@jhmi.edu (L.N.);; 2 Department of Medical and Surgical Sciences, University of Bologna, 40138 Bologna, Italy; 3 Department of Informatics, Systems, and Communication, University of Milano-Bicocca, 20126 Milan, Italy; 4 Department of Internal Medicine, Radboud University Medical Center, 6525 AJ Nijmegen, The Netherlands; 5 Department of Psychology and Cognitive Science, University of Trento, 38068 Rovereto, Italy; 6 Department of Biomedical Engineering, Johns Hopkins University, Baltimore, MD 21218, USA; 7 Institute of Biomedical Technologies, National Research Council, 20054 Segrate, Italy; 8 AccYouRate Group S.p.A., 67100 L’Aquila, Italy; 9 Department of Radiology, Johns Hopkins University, Baltimore, MD 21205, USA

**Keywords:** ECG, wearable technology, machine learning, deep learning, m-health

## Abstract

Worldwide, population aging and unhealthy lifestyles have increased the incidence of high-risk health conditions such as cardiovascular diseases, sleep apnea, and other conditions. Recently, to facilitate early identification and diagnosis, efforts have been made in the research and development of new wearable devices to make them smaller, more comfortable, more accurate, and increasingly compatible with artificial intelligence technologies. These efforts can pave the way to the longer and continuous health monitoring of different biosignals, including the real-time detection of diseases, thus providing more timely and accurate predictions of health events that can drastically improve the healthcare management of patients. Most recent reviews focus on a specific category of disease, the use of artificial intelligence in 12-lead electrocardiograms, or on wearable technology. However, we present recent advances in the use of electrocardiogram signals acquired with wearable devices or from publicly available databases and the analysis of such signals with artificial intelligence methods to detect and predict diseases. As expected, most of the available research focuses on heart diseases, sleep apnea, and other emerging areas, such as mental stress. From a methodological point of view, although traditional statistical methods and machine learning are still widely used, we observe an increasing use of more advanced deep learning methods, specifically architectures that can handle the complexity of biosignal data. These deep learning methods typically include convolutional and recurrent neural networks. Moreover, when proposing new artificial intelligence methods, we observe that the prevalent choice is to use publicly available databases rather than collecting new data.

## 1. Introduction

The electrocardiogram (ECG) is among the most commonly utilized clinical tests for patient monitoring and assessment because it is easy to acquire and provides extensive information about patients’ cardiac health [1]. Instead, continuous, real-time, remote monitoring allows for a more rigorous oversight of patients’ conditions, even compared to in-hospital observation. Wearable devices to address monitoring are now a prominent focus of industry [1,2,3,4,5,6], which in turn provides strong motivation for applying artificial intelligence (AI) algorithms to ECG signals for automated disease detection and prediction [7,8,9,10,11].

**Figure 1:**
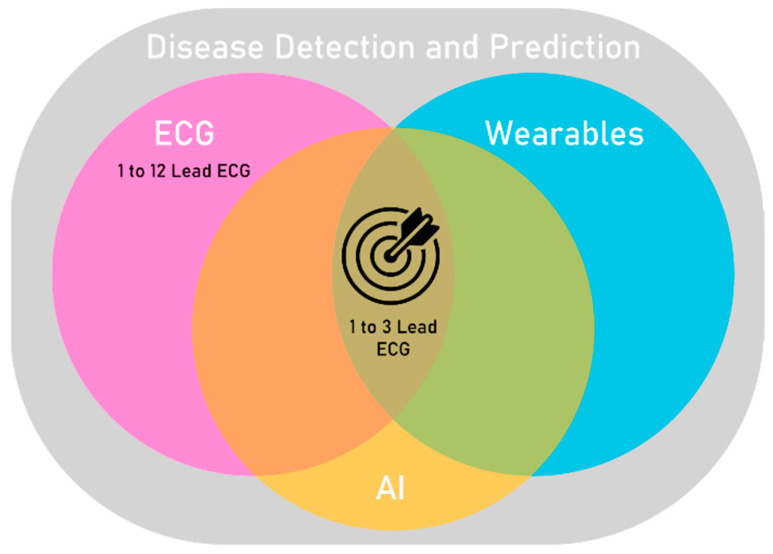
The synergy of ECG recording wearable devices and artificial intelligence algorithms enables disease detection and prediction.

Therefore, this review focuses on wearable medical devices for ECG acquisition followed by AI analysis (ECG-AI) to predict and detect specific diseases (Figure 1).

We mainly focused on the published results obtained with single-lead ECG systems, which are widely used in ambulatory monitoring but are not comfortable to wear for long periods. The use of single-lead ECG has the potential to give important diagnostic information on the user’s health [1,5] but also has some limitations compared to the standard 12-lead ECG [6].

**Figure 2:**
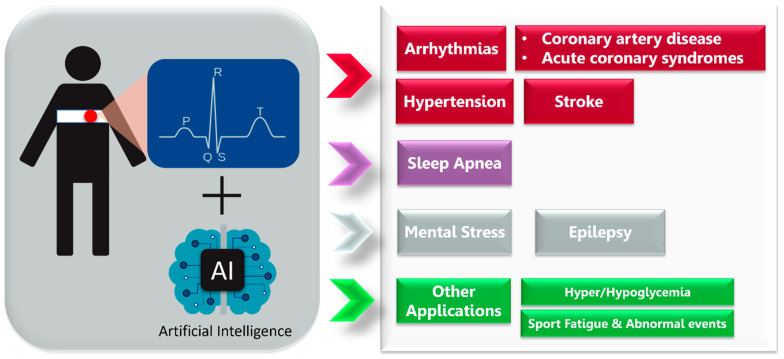
Main areas of electrocardiography- and artificial-intelligence-based medical application reviewed in the present work.

We examined publications on ECG signals and AI technology applied to wearable and mobile devices for predicting and detecting diseases. Most of the included papers are related to CVD, followed by, in order of number of published studies, the other three groups: (1) sleep apnea, (2) mental health and epilepsy, and (3) other applications such as hyperglycemia and hypoglycemia (Figure 2). While other diseases such as hyperkalemia, hypokalemia, and acute pulmonary embolism are addressed in the literature related to ECG-AI, these studies were not included here because they generally use 12-lead ECGs and do not focus on wearable applications.

## 2. Cardiovascular System

### 2.1. Diseases

ECG-based monitoring technologies with disease detection and prediction capabilities have been developed [12,13,14,15,16,17,18]. This section summarizes significant advancements related to two broad categories of cardiac conditions, namely arrhythmias and coronary artery disease.

#### 2.1.1. Arrhythmias

**Figure 3:**
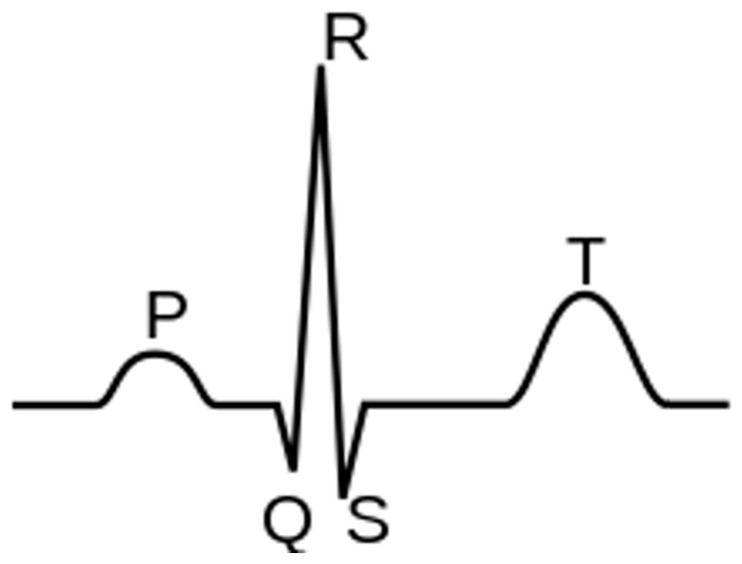
Components of a normal electrocardiogram include P- and T-waves and the QRS complex.

Cardiac arrhythmia is an abnormal rhythm of the heartbeat [19]. The electrical pathway of a normal cardiac contraction has a characteristic electrical pattern on an ECG recording, comprised of a “P” wave (indicating atrial depolarization), followed by a “QRS” complex (indicating ventricular depolarization), and a “T” wave (indicating ventricular repolarization). A typical ECG is shown in Figure 3.

Perturbations in the ECG may indicate underlying pathophysiologic changes. Common conditions that can be discerned from ECG changes include various arrhythmias. The most common type of irregular arrhythmia is atrial fibrillation (AF), which is characterized by disorganized electrical impulses of the atrium. AF increases the risk of stroke by up to 17% annually in high-risk individuals [20]. In addition, AF with sustained ventricular rates greater than 110 beats per minute can lead to cardiomyopathy, heart failure (HF), and sudden cardiac death if not adequately treated [21]. The worldwide prevalence of AF was estimated at approximately 46 million individuals in 2016 [22], with up to one-third of these individuals being asymptomatic and thus unaware they have AF while also being at increased risk of stroke.

In addition to AF, there are other arrhythmias for which wearable ECG devices are amenable including premature atrial contraction, premature ventricular contraction (PVC), atrial flutter, atrioventricular reentrant tachycardia, atrioventricular nodal reentrant tachycardia, and first-, second-, or third-degree heart block. Several recent papers demonstrated the use of wearable technology capable of identifying premature atrial contractions or PVCs with over 97% accuracy [17,18,23,24]. A class of malignant arrhythmias has a high risk of progression to cardiac arrest or even death [25]. Examples of malignant rhythms include ventricular tachycardia and ventricular fibrillation.

#### 2.1.2. Coronary Artery Disease

Coronary artery disease is the insidious buildup of cholesterol plaques within the walls of the arteries of the heart, eventually leading to a narrowing of the blood vessels [26]. When the narrowing of blood vessels surpasses a critical threshold (often described as a narrowing of greater than 70% of the inner lumen of the artery), symptoms such as exertional chest pain (angina), exertional shortness of breath, and decreased exercise tolerance can occur. Coronary artery disease accounts for the vast majority of cardiac-related deaths [27]. A diagnosis of coronary heart disease generally requires a history and physical exam, a stress test, and an observation of ECG changes suggestive of cardiac ischemia.

Various ECG changes are associated with acute and chronic ischemia. For instance, the presence of Q waves in any lead other than the right-sided leads (i.e., aVR and V1, occasionally in III) is often pathognomonic for prior infarction and non-viable myocardium [28]. On the other hand, chronically inverted T-waves and ST depressions are generally described as non-specific ECG patterns and are difficult to interpret on their own, requiring additional context. However, in the correct clinical setting, these changes can be dynamic where they appear while the patient has active symptoms and normalize when they resolve. Such dynamic changes indicate significant coronary artery disease that needs to be aggressively investigated because the sudden development of ST-segment elevation associated with symptoms suggests an evolving coronary artery occlusion and subsequent myocardial impairment. Such patients need to be examined then treated immediately. Future work to develop ECG-AI wearables for real-time detection of acute ischemia will likely improve outcomes.

### 2.2. Wearables

ECG-AI has been combined with wearable devices to investigate various cardiac pathologies, including AF, stroke, cardiac arrest, and heart failure. In fact, arrhythmia monitoring is among the most popular applications of wearable devices in medicine. However, wearable devices are limited in their ability to detect arrhythmias other than AF [6,29], particularly ventricular tachycardia or ventricular fibrillation, which is why wearable technologies capable of accurately detecting either ventricular tachycardia or ventricular fibrillation were limited in the literature.

Overall, there are a limited number of studies involving wearables. Some studies use commercially available wearables to explore the implementation of ECG-AI. For example, devices such as the Amazfit Band 1S (PPG and single-lead ECG) [30], the HealthyPiV3 biosensors [31], or Polar H7 HR monitor [32] have been utilized. A few research groups have even built their own wearable ECG recording prototypes [33,34,35].

The Food and Drug Administration (FDA) recently approved a single-lead ECG smartwatch proven to detect AF in the general population [36]. Another device developed for AF monitoring and detection includes a single-lead wireless ECG patch worn over the chest, which provides real-time ECG monitoring using cloud-based data analysis and data sharing with medical providers [13]. Similarly, a custom wrist-based wearable ECG recorder was compared to the standard 12-lead configuration via a prospective, registration-only, single-center study for the detection of AF [37]. Although a small dataset based on a relatively low number of patients was used, a sensitivity and specificity of 99.4% and 99.8%, respectively, were reported. The wrist-based device’s convenience and ease of use was highlighted as an attractive modality for arrhythmia detection in the general population. Lastly, a single-lead ECG chest belt that transmits data to a cloud service for analysis was described, and a sensitivity and specificity of 100% and 95.4%, respectively, were reported [38]. The study included a user experience questionnaire, showing that 77% of participants preferred the chest belt to a standard 3-lead Holter monitor. Additional studies detecting AF have been performed using commercially available heart rate monitors and ECG systems [30,31,32,39,40,41].

### 2.3. Algorithms

#### 2.3.1. Arrhythmia

Due to their ubiquitous availability, most ECG-AI research has been performed using public databases such as the PhysioNet [42] MIT-BIH Arrhythmia database [43,44] while only a few research groups have independently acquired data from patients. Curated and publicly available datasets include physician annotations that provide a reference for ECG-AI algorithm training (Table 1).

Machine learning (ML) and deep learning (DL) have both been extensively applied to ECG data to detect arrhythmias. Despite being relatively poorer performing, ML is utilized for arrhythmia detection due to some of the limitations of DL, including resource-intensive hyper-parameters to find the optimal network configuration and the challenges in understanding the rules underlying trained prediction models [45]. However, DL has shown modest improvements over ML for arrhythmia detection. The varying sample resolutions could pose a challenge for these techniques, but it was shown that it is possible to accurately detect arrythmias using down sampled ECG data [46].

ML approaches often include the use of decision tree ensembles such as Random Forest [13,47] or support vector machines (SVMs) [40,48] for arrhythmia classification. Multi-stage and multi-level classification systems derive local features of atrial and ventricular activity through a combination of SVMs and decision trees and global features from the raw ECG recording, ultimately leading to classification through linear SVMs. Furthermore, a rotated linear-kernel SVM has been proposed in which two SVM classifiers are trained, one on the global dataset and the other on a patient-dependent dataset obtaining two different discriminant hyperplanes. The final hyperplane, obtained by rotating the first hyperplane by a specific amount towards the second hyperplane, resulted in an improved sensitivity [49]. Similarly, this ML method has been used with a classifier of de-correlated Lorenz plots of inter-beat intervals [32], and with another classifier built on features extracted through pre-processing methods from density Poincaré plots that represented the ECG segments [23]. Alternatively, the use of SVMs through a semi-supervised learning method was demonstrated [50], while the hybrid framework effectively combined the advantages of ensemble learning and evolutionary computation to maximize arrhythmia classification accuracy [51].

With regard to DL approaches, convolutional neural network (CNN) architecture was applied to arrhythmia [52,53,54] and AF classifications [24,55]. Other architectures of interest for AF classification include a deep densely connected neural network based on 12-lead ECG [15], a feedforward neural network based on features encompassing R-R intervals [56] and another based on the Lightweight Fusing Transformer [17]. Hybrid constructions have also been presented, frequently involving an architecture based on a CNN and long short-term memory (LSTM) [57,58,59,60], as well as an extension to SVM with predictions from a CNN [41]. With a similar premise to the rotated linear-kernel SVM [49], a study has proposed a Generic CNN suitable for all individuals, and a tuned dedicated CNN as obtained by finetuning the previous model with respect to a specific individual [61]. Another approach of interest is the use of multi-scale (MS) CNNs to improve feature extraction and classification from ECG data [62]. Additionally, a global hybrid multi-scale convolutional neural network (Acc 99.84%) was proposed as an advanced alternative to other MS-based approaches through their hybrid multi-scale convolution module [63].

Previous research has also designed lightweight DL models using cloud-based applications to efficiently classify ECG data. These approaches utilize fused recurrent neural network (RNN) layers instead of standard RNN layers [39]. The application of compression [44,64] and conversion techniques (Acc 99.60%) [65], and model-hardware co-optimization [66] to reduce the model’s size in terms of computational parameters, resulted in lower memory consumption and inference time. Other techniques to accelerate arrhythmia detection include real-time data compression, signal processing, and data transmission [67,68,69]. Alternatively, ECG data may be compressed to enable real-time AF classification [70,71].

In addition to directly processing ECG data, some studies focused on its two-dimensional representation, which can be used for feature extraction and/or classification. Examples of these representations include spectrograms [31] and iris spectrograms [72]. Alternatively, the ECG signal may be transformed into an electrocardiomatrix, which is a two-dimensional representation that includes the rhythm and shape of the QRS complex [73]. A beat-interval-texture CNN was then used to process the electrocardiomatrix. In this architecture, there are four different layers: the first two layers perform low-level feature extraction, and the two subsequent layers perform high-level feature extraction using three types of convolution filters (beat, interval, and texture). Next, a feature attention layer weighs the identified features concerning the arrhythmia classes and uses such weighted features for classification.

Deep metric learning for PVC detection has also been demonstrated [18]. Such learning methods combine the mechanisms of metric learning for effective feature extraction in which the features are processed with k-nearest neighbors for binary classification. In comparing ML and DL, the former may use the ECG to define summary features that provide physiologic insight, whereas the latter automatically extracts discriminating information from complete waveforms [74]. ML and DL may complement one another, as demonstrated by the multiview fusion classification model in which both summary and deep features from ECG signals were fused [57]. However, DL may independently offer some physiologic information via gradient-weighted class activation mapping, which can highlight the relative contributions of the temporal regions of the ECG signal that most contribute to the AI-obtained classification [73].

**Table 1:** Summary of ECG-based AI algorithms applied to arrhythmias.

Authors (Year)	Specific Application	ECG System (Sampling Frequency)	AI Algorithm/Method	Database/Dataset	Performance (%)				
					Acc	Sen	Spe	AUC	F1
Jeon et al. (2020) [39]	General arrhythmias	**2-lead ECG patch [Samsung S-Patch 2]** **(256 Hz)**	Recurrent Neural Networks	MIT-BIH Arrhythmia Wearable device: S-Patch 2	99.80	-	-	-	-
Plawiak et al. (2020) [51]	General arrhythmias	-	Deep Genetic Ensemble of Classifiers	MIT-BIH Arrhythmia	99.37	94.62	99.66	-	-
Panganiban et al. (2021) [31]	General arrhythmias	**2-lead ECG [HealthyPiV3 biosensors]** **(n.s.)**	CNN	MIT-BIH Atrial Fibrillation, PAF Prediction Challenge, PTB Diagnostic ECG, Challenge 2015 Training Set, Fantasia, and PAF Prediction Challenge. ECG signals collected for this study	98.73	96.83	99.21	-	96.83
Alqudah et al. (2021) [72]	General arrhythmias	-	CNN	IEEE DataPort MIT-BIH Arrhythmia	99.13	99.31	99.81	-	-
Yildirim et al. (2018) [52]	General arrhythmias	-	CNN	MIT-BIH Arrhythmia	95.20	93.52	99.61	-	92.45
Bazi et al. (2020) [40]	General arrhythmias	**Wireless 3-lead ECG sensor [Shimmer Sensing** **(100, 200 Hz)**	SVM	12-lead Tech-Patient CARDIO ECG simulator Wearable device: Shimmer Sensing MIT-BIH Arrhythmia	95.10	95.80	-	-	-
Lee et al. (2022) [44]	General arrhythmias	-	CNN	ECG from patients at the Korea University Anam Hospital in Seoul, Korea	97.90	98.30	97.60	99.70	97.70
Itzhak et al. (2022) [46]	General arrhythmias	-	Random Forest	Annotated Holter ECG database acquired at the University of Virginia Heart Station	93.30	91.30	81.30	95.30	90.60
Li et al. (2018) [61]	General arrhythmias	-	Generic CNN and Tuned Dedicated CNN	MIT-BIH Arrhythmia	96.89	-	-	-	-
Ran et al. (2022) [66]	General arrhythmias	12-lead ECG prototype (500Hz)	Deep CNN	12-lead ECG recordings from three centers of Tongji Hospital	-	89.10	99.70	94.40	91.30
Ribeiro et al. (2022) [65]	General arrhythmias	-	CNN	MIT-BIH Arrhythmia	99.60	98.50	99.80	-	98.80
Hua et al. (2018) [50]	General arrhythmias	-	SVM	MIT-BIH Arrhythmia	98.58	97.70	99.62	-	-
Karthiga et al. (2021) [53]	General arrhythmias	-	CNN	MIT-BIH Arrhythmia	91.92	90.21	95.19	-	90.11
Zhang et al. (2022) [54]	General arrhythmias	-	CNN	MIT-BIH Arrhythmia	98.74	98.11	99.05	-	-
Lee et al. (2021) [73]	General arrhythmias	-	Beat-Interval-Texture CNN	2017 PhysioNet/Computing in Cardiology Challenge	-	80.73	-	-	81.75
Smisek et al. (2018) [48]	General arrhythmias	-	SVMs Decision Tree	2017 PhysioNet/Computing in Cardiology Challenge	-	-	-	-	81.00
Shin et al. (2022) [58]	General arrhythmias	-	CNN-Bidirectional Long Short-Term Memory	MIT-BIH Arrhythmia	91.70	92.00	91.00	99.40	92.00
Alqudah et al. (2021) [75]	General arrhythmias	-	CNN	MIT-BIH Arrhythmia	93.80	95.20	97.40	-	93.60
Huang, et al. (2021) [57]	General arrhythmias	-	CNN-LSTM	MIT-BIH Arrhythmia	98.93	96.46	99.33	-	-
Tang et al. (2019) [49]	General arrhythmias	-	SVM	MIT-BIH Arrhythmia	98.90	92.80	99.40	-	92.00
Sakib et al. (2021) [64]	General arrhythmias	-	Deep-Learning-based Lightweight Arrhythmia Classification (CNN)	MIT-BIH Supraventricular Arrhythmia MIT-BIH Arrhythmia St Petersburg INCART 12-lead Arrhythmia Sudden Cardiac Death Holter	96.67	-	-	97.96	-
Shao et al. (2020) [13]	AF	**Custom 1-lead ECG patch** **(250 Hz)**	Decision Tree Ensemble	2017 PhysioNet/Computing in Cardiology Challenge MIT-BIH Atrial Fibrillation Simulated ECG signals from generator FLUKE MPS450	99.62	99.61	99.64	-	92.00
Chen et al. (2020) [30]	AF	**PPG & 1-lead ECG [Amazfit Health Band 1S]** **(250 Hz)**	CNN	PPG and single-channel ECG data	94.76	87.33	99.20	-	-
Cai et al. (2020) [15]	AF	12-lead ECG (500 Hz)	Deep Densely connected Neural Network	12-lead ECG 10s recordings collected from multiple hospitals and wearable ECG devices (3 different data sources)	99.35	99.19	99.44	-	-
Cheng et al. (2020) [70]	AF	-	Deep Learning Neural Networks	MIT-BIH Atrial Fibrillation	97.52	97.59	97.40	-	98.02
Fan et al. (2018) [62]	AF	-	Multi-Scale CNN	2017 PhysioNet/Computing in Cardiology Challenge	98.13	93.77	98.77	-	-
Ramesh et al. (2021) [55]	AF	-	CNN	Train: MIT-BIH Normal Sinus Rhythm, MIT-BIH Atrial Fibrillation, MIT-BIH Arrhythmia Test: UMass PPG, acquired from wrist-worn wearable devices	95.50	94.50	96.00	95.30	93.40
Ma et al. (2020) [41]	AF	**SmartVest system** **(400 Hz)**	SVM extended with CNN predictions	Train: MIT-BIH Atrial Fibrillation Test: PhysioNet/Computing in Cardiology Challenge 2017, China Physiological Signal Challenge (CPSC) 2018, 24-h ECG recording (12 h before and 12 h after the radio frequency ablation surgery) collected from an AF patient with the wearable device	99.08	98.67	99.50	-	-
Lown et al. (2020) [32]	AF	1. 12-lead ECG **(n.s.)** **2. HR monitor [Polar H7 (PH7) HR]** **(n.s.)**	SVM	MIT-BIH Atrial Fibrillation MIT-BIH Arrhythmia	-	100.0	97.60	-	-
Zhang et al. (2021) [63]	AF	-	Global Hybrid Multi-Scale Convolutional Neural Network	China Physiological Signal Challenge 2018 (12-lead ECG) 2017 PhysioNet/Computing in Cardiology Challenge (single-lead ECG)	99.84	99.65	99.98	-	99.54
Zhang et al. (2020) [71]	AF	-	CNN	MIT-BIH Atrial Fibrillation	96.23	95.92	96.55	-	96.25
Chen et al. (2022) [56]	AF	-	Feedforward Neural Network	2017 PhysioNet/Computing in Cardiology Challenge MIT-BIH Arrhythmia	84.00	84.26	93.23	89.40	-
Mei et al. (2018) [47]	AF	-	Baggin Trees	2017 PhysioNet/Computing in Cardiology Challenge	96.60	83.20	98.60	-	-
Wu et al. (2020) [45]	AF	-	Extreme Gradient Boosting	2017 PhysioNet/Computing in Cardiology Challenge MIT-BIH Atrial Fibrillation MIT-BIH Normal Sinus Rhythm MIT-BIH Arrhythmia	95.47	94.59	96.40	-	95.56
Bashar et al. (2021) [23]	AF, PAC and PVC	-	SVM	Medical Information Mart for Intensive Care (MIMIC) III	97.45	98.99	95.18	-	-
Yu et al. (2021) [18]	PVCs	-	Deep Metric Learning K-Nearest Neighbors	MIT-BIH Arrhythmia	99.70	97.45	99.87	-	-
Wang (2021) [24]	PVCs	-	CNN with improved Gated Recurrent Unit network	MIT-BIH Arrhythmia China Physiological Signal Challenge 2018	98.30	98.40	98.20	-	-
Meng et al. (2022) [17]	PVC, SPB	-	Lightweight Fussing Transformer with LightConv Attention	The 3rd China Physiological Signal Challenge 2020	99.32	92.44	-	-	93.63
Khan et al. (2020) [33]	CVDs	-	SVM	Cleveland Heart Disease dataset from the UCI repository	93.33	94.29	92.73	-	-
Dami et al. (2021) [76]	CVDs	-	LSTM Deep Belief Network	Four databases: DB1—KAGGLE heart disease dataset|DB2—Shahid Beheshti Hospital Research Center|DB3—Physionet site—Hypertensive patients|DB4—UCI Heart Disease dataset	88.42	85.13	85.54	-	-
Khan et al. (2020) [77]	CVDs	**Custom 1-lead ECG** **(n.s.)**	Deep Convolutional Neural Network	UCI machine learning repository, Framingham, and Public Health Dataset	98.20	97.80	92.80	-	95.00
Tan et al. (2021) [60]	CVDs and COVID-19	-	CNN-LSTM	MIT-BIH Arrhythmia	99.29	97.77	99.53	-	-
Mazumder et al. (2021) [59]	VT and VF	-	CNN-LSTM	MIT-BIH Malignant Ventricular Arrhythmia (VFDB) Creighton University Ventricular Tachycardia (CUDB)	-	99.21	99.68	-	-

Notes: Bold type highlights the wearable device when present and used to collect data. The best AI model/algorithm and results, when different models/algorithms, datasets, signals, and events are considered, were reported. Abbreviations: AF = atrial fibrillation; CVD = cardiovascular disease; PAC = premature atrio ventricular contractions; PVC = premature ventricular contraction; VF = ventricular tachycardia; VF = ventricular fibrillation; SPB = supraventricular premature beat; ECG = electrocardiogram; PPG = photoplethysmography; n.s. = not specified; HR = heart rate; CCN = convolutional neural network; LSTM = long short-term memory; SVM = support vector machine; Acc = accuracy; Sen = sensitivity; Spe = specificity; AUC = area under the curve of receiver-operating characteristic curves.

#### 2.3.2. Other Cardiovascular Diseases

Other cardiovascular conditions amenable to ECG-AI include myocardial infarction and heart failure (Table 2). Particularly with myocardial infarction detection, there has been a shift from ML techniques towards DL techniques [16,35,78] due to their higher performances and the fact that no handcrafted feature extraction is required. DL techniques for myocardial infarction detection include the application of both simple and complex models. Examples of simple DL models include an artificial neural network with only three layers (Acc 99.10%) [79] and CNN [12,16] and LSTM [80] algorithms. More complex DL models include a deep belief network for unsupervised heart rate variability (HRV) feature extraction and selection with LSTM for classification [76], a multi-channel lightweight model for the simultaneous analysis and classification of four ECG leads [81], and a two-dimensional CNN for the classification of ECG waveform snapshots [34]. It is important to notice that the ECG-AI determination of myocardial infarction commonly involves 12-lead data because the different leads represent different projections of the heart’s electrical activity, which is necessary to capture region-specific ischemia [12,16,78,79,80,81]. However, some algorithms were assessed based on data recorded from wearable single-lead devices [34,35].

**Table 2:** Summary of ECG-based AI algorithms applied to other cardiovascular diseases.

Authors (Year)	Specific Application	ECG System (Sampling Frequency)	AI Algorithm/Method	Database/Dataset	Performance (%)				
					Acc	Sen	Spe	AUC	F1
Gibson et al. (2022) [12]	Myocardial Infarction	-	CNN	Latin America Telemedicine Infarct Network (LATIN)	90.50	86.00	94.50	-	-
Baloglu et al. (2019) [16]	Myocardial Infarction	-	CNN	PTB ECG: MI on standard 12-lead ECG data	99.78	99.80	-	-	-
Cho et al. (2021) [82]	Heart Failure	12-lead ECG [Page Writer Cardiograph—Philips] (500 Hz)	Short-time Fourier transform–CNN combination	ECG from multicenter study	82.50	92.10	82.10	92.90	-
Wasimuddin et al. (2021) [34]	Myocardial Infarction	**Custom 1-lead ECG** **(n.s.)**	CNN	European ST-T Custom wearable device	99.26	99.27	99.27	-	-
Chowdhury et al. (2019) [35]	Myocardial Infarction-Cardiac Arrest	**Custom 1-lead ECG** **(500 Hz)**	Support Vector Machine	MIT-BIH ST Change Normal subjects and an ECG simulator to simulate abnormal ST-elevated MI situations to test the functionality of the complete system in real-time	97.40	99.10	-	-	98.70
Shahnawaz et al. (2021) [79]	Myocardial Infarction	-	Artificial Neural Network	PTB (PhysioNet)	99.10	100.00	98.10	-	99.00
Sopic, et al. (2018) [78]	Myocardial Infarction	-	Random Forest	PTB (PhysioNet)	80.30	87.95	79.63	-	-
Martin et al. (2021) [80]	Myocardial Infarction	-	Deep Long Short-Term Memory	PTB-XL and PTB (PhysioNet)	79.69	76.59	85.89	-	83.42
Cao et al. (2021) [81]	Myocardial Infarction	-	Multi-Channel Lightweight model	PTB (PhysioNet)	96.65	94.30	97.72	96.71	-

Notes: Bold type highlights the wearable device when present and used to collect data. The best AI model/algorithm and results, when different models/algorithms, datasets, signals, and events are considered, were reported. Abbreviations: n.s. = not specified; CNN = convolutional neural network; Acc = accuracy; Sen = sensitivity; Spe = specificity; AUC = area under the curve of receiver-operating characteristic curves.

The analysis of 12-lead data also enabled the screening of heart failure with reduced ejection fraction (Acc 82.50%) [82]. Following a short-time Fourier transform in combination with a CNN, an interpretable model highlighted the essential regions in the various ECG leads associated with the final classification. In particular, the lateral (aVL, I, −aVR, V5, V6) and anterior leads (V3, V4) greatly impacted heart failure with a reduced ejection fraction detection. In contrast, the performance of the inferior leads (II, aVF, III) was relatively poor. The findings also confirmed that a rightward T-wave axis, prolonged QT duration, and prolonged QTc are associated with heart failure and that the T-wave axis is an independent and strong risk factor for cardiac events in the elderly.

## 3. Sleep Apnea

**Figure 4:**
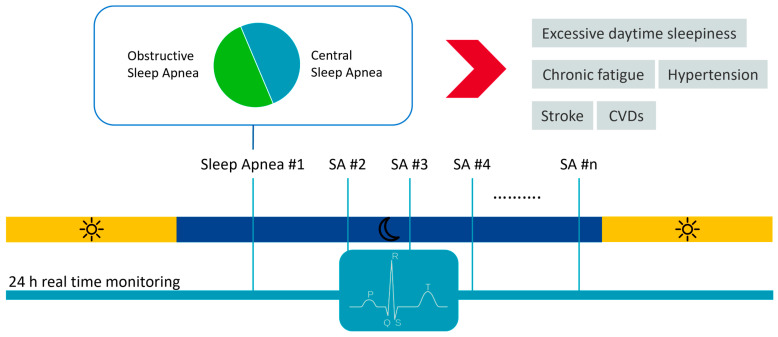
Sleep apnea and its consequences relative to diagnostics potentially enabled by continuous real-time ECG monitoring.

Sleep apnea is a sleep disorder characterized by the interruption of breath during sleep [83]. It is divided into two subtypes: central sleep apnea (CSA) and obstructive sleep apnea (OSA). (Figure 4). CSA is less prevalent and results from the abnormal regulation of breathing in the brainstem respiratory centers, which leads to an absence of or diminution in involuntary respiratory effort while asleep [84]. OSA is a highly prevalent sleep-related disorder characterized by the repetitive complete obstruction (apnea) or partial obstruction (hypopnea) of the upper airway that results from loss of muscle tone in anatomically susceptible persons [85]. It is estimated that OSA affects almost 1 billion people globally [86], with 425 million adults aged 30–69 years having moderate to severe OSA [87]. CSA is associated with heart failure, renal failure, and the acute phases of stroke, while OSA can lead to excessive daytime sleepiness, chronic fatigue, hypertension, stroke, and other cardiovascular disorders. Thus, early and accurate diagnosis of sleep apnea is essential.

Laboratory-based polysomnography has been used as a reference standard for diagnosing OSA. Polysomnography involves the overnight recording of: the bilateral occipital, central, and frontal electroencephalogram; chin, leg, and surface electromyogram; left and right eye electro-oculogram; and ECG, pulse-oximetry, airflow, and respiratory effort. Yet, polysomnography is time-consuming, expensive, and uncomfortable for the patient and requires a trained technician. Therefore, an ECG-AI approach to sleep apnea diagnosis is a potentially convenient and cost-effective alternative [88].

### 3.1. Wearables

To our knowledge, no studies have investigated the use of wearable ECG-AI devices for sleep apnea detection. In fact, sleep apnea ECG data analysis has solely relied on existing datasets such as the PhysioNet Apnea-ECG database [89] or by collecting new data based on polysomnography.

### 3.2. Algorithms

When automatically identifying OSA from ECG recordings, DL is preferable over traditional ML because of its ability to automatically learn discriminating features from raw data (Table 3). For instance, a CNN using a modified LeNet-5 architecture was compared against five conventional approaches [90]. The superior performance of CNN (Acc 96.00%) for OSA classification was further reinforced by the finding that short-term (30 s) ECG segments were classified into four (normal, mild, moderate, and severe) versus two (normal and OSA) categories [91].

An OSA detection framework based on a multiscale dilation attention CNN and a weighted loss time-dependent classification model for feature extraction and classification were proposed to fully exploit ECG information via DL [92]. The novelty of the multiscale dilation attention one-dimensional CNN lies in the parallel multi-branch structure and dilation operations, which allow the model to explore the feature space efficiently by assigning feature weight with the efficient channel attention module. The classifier addresses the challenges following temporal dependence between ECG segments using a weighted loss function that reduces class imbalance.

Hybrid DL methods have also been proposed in which different methods are combined. Examples are the CNN and LSTM combination with SVM [93], a hybrid three-dimensional CNN-LSTM combination where 20 successive single segments were analyzed simultaneously to include the time evolution pattern of the ECG [94], and a CNN representation learning model for feature extraction combined with a temporal dependence model for classification [95]. To address the limited ability of classic network architectures in feature extraction, the use of a one-dimensional squeeze-and-excitation residual group network to detect OSA using inter-beat intervals and R-wave and Q-wave amplitude from two-minute ECG signal segments was proposed [96]. The network architecture is a CNN in which the residual group convolutions are included to alleviate the computational burden whereas the squeeze-and-excitation mechanism manages the importance of the three inputs.

**Table 3:** Summary of ECG-based AI algorithms applied to sleep apnea.

Authors (Year)	ECG System (Sampling Frequency)	AI Algorithm/Method	Database/Dataset	Performance				
				Acc	Sen	Spe	AUC	F1
Bahrami et al. (2022) [94]	-	Hybrid three-dimensional CNN—LSTMs	Apnea-ECG (PhysioNet)	94.95	93.92	95.63	-	93.65
Yang et al. (2021) [96]	-	Squeeze-and-excitation residual group network	Apnea-ECG and UCDDB dataset (PhysioNet)	90.30	87.60	91.90	96.50	87.30
Urtnasan et al. (2020) [91]	-	CNN	Subjects studied with overnight PSG	96.00	-	-	99.00	99.00
Qin et al. (2022) [95]	-	CNN—Representation Learning model and Temporal Dependence model	Apnea-ECG (PhysioNet) In-group database from The Sixth Affiliated Hospital of Sun Yat-sen University	91.10	88.90	92.40	97.00	88.30
Almutairi et al. (2021) [93]	-	CNN-LSTMs and Support Vector Machine	Apnea-ECG (PhysioNet)	90.20	91.24	90.36	-	92.76
Shen et al. (2021) [92]	-	MultiScale Dilation Attention—CNN and Weighted Loss Time-Dependent	Apnea-ECG (PhysioNet)	89.40	89.80	89.10	96.40	86.60
Wang et al. (2018) [90]	-	CNN	Apnea-ECG and UCDDB dataset (PhysioNet)	87.60	83.10	90.30	95.00	-

Notes: Bold type highlights the wearable device when present and used to collect data. The best AI model/algorithm and results when different models/algorithms, datasets, signals, and events are considered were reported. Performance is reported per-segment. Abbreviations: CNN = convolutional neural network; LSTM = long short-term memory; Acc = accuracy; Sen = sensitivity; Spe = specificity; AUC = area under the curve of receiver-operating characteristic curves.

## 4. Mental Health and Epilepsy

Another field of ECG-AI application is clinical psychophysiology, which has used cardiovascular indicators for decades as proxies of cognitive and emotional processes [97]. The stress response is the most investigated of such processes and is characterized by a set of physiologic changes, including increased heart and respiratory rates, skin conductance, cortisol secretion, and muscular and pupillary dilation [98]. The individual tendency to be either hyper- or hypo-reactive is associated with an increased risk of cardiovascular disease and other somatic and mental health conditions [99,100,101]. Consequently, clinical psychophysiology aims to identify objective signs and early biomarkers of somatic and mental illness [102] with applications ranging from cardiovascular rehabilitation to clinical monitoring and work-related health and safety [103,104,105].

**Figure 5:**
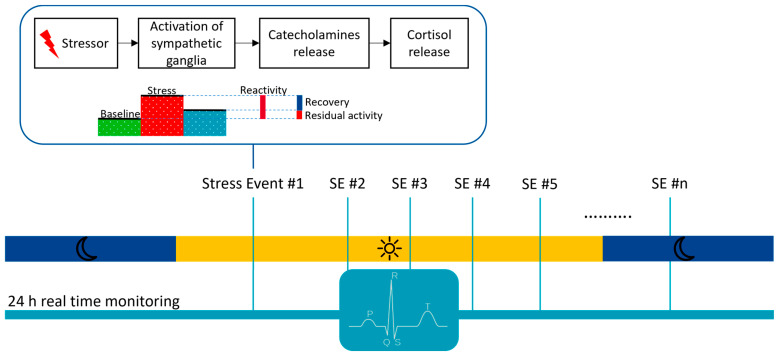
Stress response and its physiology relative to diagnostics potentially enabled by continuous, real-time ECG monitoring.

The data-gathering approach in this research field commonly entails the psychophysiological assessment, during which study participants are exposed to stressful tasks (e.g., mental arithmetic, cold pressure test, public speech) preceded by a baseline phase and followed by a recovery phase [106] (Figure 5). Such an evaluation is most widely implemented in a laboratory setting; however, several variants have been proposed to improve its everyday validity, including virtual-reality-based studies [107] and ambulatory assessments [108].

Regardless of the specific focus on stress or emotions, most of the reviewed studies (see Table 4) focused on HRV features. HRV is an index of cardiovascular flexibility and adaptability with higher HRV being associated with more effective responsivity to stressors and recovery in stress-free conditions [109]. Moreover, vagal tone is a main determinant of resting-state HRV levels, and it is also associated with a network of structures involved in emotion regulation (e.g., the amygdala) and executive functions (e.g., the prefrontal cortex) [109,110]. Therefore, HRV is among the physiologic indicators of stress, emotions, and other self-regulatory processes [111]. HRV indices in both the time and the frequency domains are widely used for ECG-AI stress detection and emotion recognition [104].

HRV is also implicated in other neuropsychologic conditions such as epilepsy and epileptic seizures, the prediction of which has profound clinical utility [112]. For instance, epileptic patients are characterized by lower high-frequency HRV and overall sympathovagal imbalance [113], and cardio acceleration (tachycardia), with HRV reductions being typical peripheral concomitants of epileptiform electroencephalography (EEG) activity [113,114].

### 4.1. Wearables

Several commercial wearable devices were used to collect ECG data for research involving stress detection and emotion recognition, including the Zephyr BioHarness 3.0 [74,115], T-REX TR100A [116], and “LaPatch” [117]. However, the continued development of public, disease-dedicated databases, such as the PhysioNet Driver stress dataset [118], allows for algorithm development and evaluation without collecting data [119]. Such an approach is mainly used for epilepsy applications, where the condition is monitored and not induced and where the biosignals are directly evaluated from patients to detect and predict event occurrence. In these studies, ECG and EEG data are analyzed together. An additional two studies were reported in which the ECG signal was collected with ad-hoc wearable prototypes alongside other biosignals such as the EEG [120,121].

**Table 4:** Summary of ECG-based AI algorithms applied to mental health and epilepsy.

Authors (Year)	Specific Application	ECG System (Sampling Frequency)	AI Algorithm/ Method	Sample/Database	Protocol/Tasks	Performance (%)				
						Acc	Sen	Spe	AUC	F1
Seo et al. (2019) [74]	Mental stress recognition	Zephyr BioHarness (n.s.)	Deep Neural Network (Deep ECG-Respiration Network)	18 healthy adults	Four randomized 5 min stress tests (math or Stroop) with varying difficulty (easy vs. hard), each followed by 5 min recovery	83.90	-	-	92.00	81.00
Cho et al. (2019) [116]	Stress recognition	Training: None Testing: T-REX TR100A (256 Hz)	Deep Neural Network with transfer learning	Training: Driver stress database on PhysioNet Testing: 17 individuals	Training: 15 min resting, 20 to 60 min driving, 15 min resting Testing: 5 min baseline, 5 min simple math, 5 min recovery, 5 min hard math	90.19	93.00	85.40	93.80	92.20
Betti et al. (2017) [115]	Mental stress monitoring	Zephyr BioHarness (250 Hz)	Support Vector Machine	12 healthy individuals	10 min resting, 15 min stress tests (cold pressure and math), 10 min recovery	86.00	84.00	90.00	-	-
Huang et al. (2018) [117]	Mental fatigue detection	“LaPatch” (250 Hz)	K-Nearest Neighbors and others	29 healthy individuals	10 min resting, 10 min quiz	74.50	-	-	74.00	-
Sepulveda et al. (2021) [119]	Emotion recognition	-	Ensemble Bagged Tree and others	2018 AMIGOS	16 short videos (<250 s) and 4 long videos (>14 min)	90.30	-	-	-	89.50
Yamakawa et al. (2020) [120]	Epileptic seizure prediction	**Custom telemeter based on portable ECG** **(1 kHz)**	Multivariate Statistical Process Control	Model construction: 15 refractory epilepsy patients Model evaluation: 7 focal epilepsy patients, 7 healthy controls	Patients: 32 to 105 h ECG and video-EEG monitoring during seated or supine resting Controls: 5 to 11 h ECG ambulatory monitoring	-	85.70	-	-	-
Vandecasteele et al. (2021) [121]	Multimodal epileptic seizure detection	**1-lead ECG** **(n.s.)**	Multimodal integrating SVM (EEG) and Random Forest (ECG) predictions	135 focal epilepsy patients from the SeizeIT1, Epilepsiae- Freiburg, and Epilepsiae- Paris	Long-term pre-surgical monitoring	-	92.00	-	-	-

Notes: Bold type highlights the wearable device when present and used to collect data. The best AI model/algorithm and results when different models/algorithms, datasets, signals, and events are considered were reported. Abbreviations: ECG = electrocardiogram; n.s. = not specified; EEG = electroencephalogram; Sen = sensitivity; Spe = specificity; Acc = accuracy; AUC = area under the curve of receiver-operating characteristic curves.

### 4.2. Algorithms

Various ML and DL approaches are used in psychophysiological research. Conventional ML techniques were adopted for mental fatigue detection and emotion classification [117,119]. In particular, a wavelet scattering algorithm was successfully applied to extract more complex ECG features than the standard time- and frequency-based features [119].

ML and DL are mainly used for stress detection, as in a study where stress level was estimated through a combination of principal component analysis for feature extraction and SVM for classification [115]. Moreover, a two-branched deep learning neural network (DNN) based on the deep ECG net structure was proposed [74]. Two branches are devoted to feature extraction of ECG and respiratory features, respectively, after which they are concatenated for classification. Of interest here are the author’s visualizations of the network’s learning process, which provide insight into the network’s decision-making. In a second DNN, two training methods were investigated: training from scratch and transfer learning [116]. In the latter method, the pre-trained model parameters were determined following training on one database after which they were adjusted using a second database. Classification performance analyses indicated that the transfer learning application improved the scores of all metrics (Acc 90.19%). ECG-AI algorithms for mental stress and emotion detection are typically trained on signal segments classified as “stressed” vs. “unstressed” based on the experimental phase of the psychophysiological assessment (i.e., stressor versus baseline/recovery) [74,115,116,117], whereas one study labeled the segments based on self-report measures [117], and another studies ECG activity with changes in criterion variables such as salivary cortisol [115] and an expert rating of participants’ facial expressions [119] (see Table 4).

For seizure detection, two different ML approaches were reported. The application of a multivariate statistical process control was demonstrated via a technique that searches for changes in HRV indices that could indicate seizures [120]. Nonetheless, the system had a sensitivity of 85.7% with a false alarm rate of 0.62 times per hour, implying a need for improvement. The use of two singular models were evaluated: the first, based on SVM, to classify EEG signals and the second, based on random forest, to classify ECG signals [121]. The classifiers were used against a multimodal model by integrating the predictions of the two models for seizure detection. Performance evaluation showed that integrating the prediction results of both physiologic signals in the multimodal model increased sensitivity while maintaining the same false alarm rate for two out of three databases. These studies typically used data from long-term pre-surgical monitoring [120,121,122]. The AI algorithms were then trained and tested against expert annotation of video-recorded EEG segments, which were categorized as during, after, or between seizures. Overall, these studies were characterized by lower heterogeneity in terms of research protocols and reported algorithm performance metrics compared to stress and emotion recognition studies due to the higher availability of research standards for clinical validation [123].

Studies involving ECG-AI wearables to detect mental health conditions are limited in several ways. Firstly, many studies used small samples with poorly specified or even unspecified inclusion criteria. Such low statistical power limits algorithm performance, reproducibility, and the generalizability of results. Secondly, signal pre-processing steps, including the detection of ECG components, artifact identification, and computation of the ECG features, are substantially different among the reviewed studies. Some studies used ECG tracing of 20 s or less, which excludes the use of HRV features such as the low frequency power (requires a frequency of 0.04 Hz or oscillations as long as 25 s) because signal segments lasting at least 10 times the lower frequency bound (about 4 min) have been recommended to provide proper estimates [124].

## 5. Other Applications

Examples of ECG-AI applied to other areas are reported in Table 5. Applications include the evaluation of blood sugar and sports medicine.

### 5.1. Wearables

Public databases relating to ECG and outcome data are currently available for the most common cardiac conditions such as AF and only a minority are available for other diseases. Therefore, consumer devices such as the Medtronic Zephyr BioPatch™ HP80 [125] and single-lead ECG prototypes [126,127] have been used to collect patient-specific data related to other medical conditions for subsequent AI analysis. However, the number of publicly available datasets for tailored medical applications is increasing [42].

**Table 5:** Summary of other applications of ECG-based AI algorithms.

Authors (Year)	Specific Application	ECG System (Sampling Frequency)	AI Algorithm/Method	Database/Dataset	Performance (%)				
					Acc	Sen	Spe	AUC	F1
Cordeiro, et al. (2021) [126]	Blood Sugar-Hyperglycemia	**Custom 1-lead ECG with Analog AD-8232** **(1000 Hz)**	Deep Learning Neural Network	60 s ECG, Blood glucose, and other profile information (such as age, gender, height, weight, and heart rate)	-	87.57	85.04	94.53	-
Porumb et al. (2020) [125]	Blood Sugar-Hypoglycemia	**1-lead ECG [Medtronic Zephyr BioPatch™ HP80** **(250 Hz)**	Convolutional Neural Networks + Recurrent Neural Networks	ECG signals and actigraphy, recorded continuously during a nominal period of 14 nights for each subject. 8 healthy participants were recruited: 4 hypoglycemic and 4 healthy	90.00	88.30	92.20	-	-
Luo (2020) [127]	Sport-Fatigue & Abnormal Events	**Smart wearable device [based on OpenBCI]** **(n.s.)**	Weighted one-class SVM	5400 sub-signals from 30 volunteers during 1 h	93.65	-	-	96.70	-

Notes: Bold type highlights the wearable device when present and used to collect data. The best AI model/algorithm and results when different models/algorithms, datasets, signals, and events are considered were reported. Abbreviations: n.s. = not specified; SVM = support vector machine; Acc = accuracy; Sen = sensitivity; Spe = specificity; AUC = area under the curve of receiver-operating characteristic curves.

### 5.2. Algorithms

ECG-AI has been successfully used to detect hyperglycemia and hypoglycemia [125,126]. A novel feature extraction method and a ten-layer artificial neural network classifier for the detection of hyperglycemia [126] was proposed, and it achieved an improvement of 53% versus the previous models. A person-specific system, including a DL model for each participant, was proposed for the detection of hypoglycemia [125]. Specifically, the data recorded from the first few days were used for training, while the rest was used for system evaluation. Two models were investigated: a CNN and a CNN—RNN combination. The CNN module produced a fixed-length ECG to be further processed by the next RNN module.

Another application of ECG-AI is in sports medicine to evaluate fatigue and abnormal health events in real-time. The effectiveness of this approach was demonstrated via a weighted one-class SVM using signals recorded on volunteers undergoing specific tasks [127].

## 6. General Challenges and Limitations

The clinical reliability of wearable devices is challenged by several factors including the fact that mobile versions collect fewer data compared to their clinical analogs. An example is that the ECGs of wearable devices are typically single to triple leads, while those utilized clinically feature twelve leads. Wearable technologies are also intended to be worn throughout the activities of daily living, which results in an increased likelihood of collecting intermittent or noisy data. Furthermore, the real-time effectiveness of corresponding AI algorithms are potentially compromised by processing demands relative to battery capacity or, when the processing is to be carried out on the cloud, limited connection to wireless networks in rural areas.

Once recorded via wearable devices, data are commonly reviewed by physicians when such information would be valuable in order to better understand a patient’s history [128]. However, diagnoses and predictions provided by AI algorithms are less readily accepted by clinicians [129] because the basis for these decisions is a black box. That is, an AI algorithm may decide on a particular medical condition, but the inherent lack of physiologic insight makes the reliability of such decisions uncertain by clinical standards. Determinations made by supervised AI algorithms are therefore more likely to be clinically acceptable if more insight into the physiologic mechanism by which they make their predictions can be provided.

Two limitations result from the need to provide physiologic detail. Firstly, defining summary domain-aware features to enable supervised AI reduces the dimensionality of the data and may thus limit the prediction potential at the expense of a better physiologic understanding. Indeed, to perform supervised learning and therefore satisfy clinical standards for physiologic understanding, data should be processed to obtain translatable summary features. Regarding ECG analysis, such characteristics may include the R–R interval, QRS width and magnitude, and ST-segment elevation or depression, among others. Nonetheless, this approach relies on knowing what summary features to define and doing so comprehensively. Unfortunately, the definition of translatable characteristics relies on those that are already known via traditional medicine. These characteristics are the most obvious to human interpretation, which thus undermines the main advantage of using AI: the ability to make determinations beyond the threshold of human elucidation. Secondly, it may also be desirable to perform processing steps such as truncating, filtering, or down-sampling data to make physiologic detail more obvious or optimize input before initiating an AI algorithm. However, these steps also potentially remove valuable information beyond the level of human interpretation. In moving towards a compromise of deeper knowledge with some physiologic insight, heat maps that highlight the temporal segment of the ECG most influential in making a classification are valuable [73].

Another challenge facing the clinical adoption of AI diagnoses is that there are no standards for defining what level of correctness is sufficient to replace a physician as the primary assessor. Such a threshold is particularly important to consider in the context of AI algorithms being trained by physician specialists because AI diagnoses are then relative to the most expert clinical standard rather than the average [128]. Additionally, this standard assumes that all patients have access to the best physician specialist with whom the AI algorithm is being compared. In fact, many individuals may not have any access at all, especially in real-time. Thus, wearable devices in conjunction with AI algorithms offer far greater monitoring of patients but have a higher standard for diagnostic reliability.

An additional limitation of current AI methods is that algorithm training requires the availability of quality data. In most cases, such datasets need to be large enough to be divided into training and testing sets while also being curated so that most fields are complete and are purged of erroneous information. As shown in this review, many publicly available, condition-specific datasets are emerging. However, developers should keep in mind that each database has its limitations (e.g., not socioeconomically or racially diverse enough) that narrow the database’s scope of use. After the acceptance of an algorithm, ongoing post-application clinical validation is essential to maintaining confidence in diagnostic or predictive correctness but is more challenging because these data are not curated and may thus be noisy, discontinuous, or otherwise incomplete.

As demonstrated in the tables of this review, there are no standards for defining correctness, and therefore, the direct comparison of various AI methods is often not possible. However, all measures of correctness (total error rate, positive predictive value, accuracy, sensitivity, specificity, AUC, and F1) rely on base variables including true positives, true negatives, false positives, and false negatives [130,131]. The consistent reporting of all base variable values or all measures of correctness would overcome this current limitation.

In general, the methods proposed in the literature are not easy to compare due to the different datasets used in the experiments and different research targets. The most promising algorithm for ECG applications is the deep learning CNN architecture. However, in arrhythmias detection and classification, it is possible to have a clearer understanding and insight of the algorithms’ performances. In fact, arrythmia detection is a common outcome for ECG-AI technology because arrhythmias can be relatively easily identified using one-lead ECG without the need of the standard twelve-lead ECGs, making these detection techniques easier to transfer and deploy on wearable devices. Unsurprisingly, the most popular application of wearable devices in medicine are arrhythmia detectors/monitors. For the other applications, more research and datasets need to be analyzed. Based on our work, we expect an increase in interest in the applications of wearables and ECG-AI in sleep apnea and mental stress. Moreover, new applications of ECG-AI for other conditions, such as hyper/hypoglycemia, will likely see an increase in data and research work as well. Promoting challenges between research groups seems to be the best way to boost the development of the best AI solutions. Examples of such competitions include MIT-BIH Arrhythmia or the 2017 PhysioNet/Computing in Cardiology Challenge.

## 7. Towards the Future

Wearable devices will continue to have an increasing role in personalized healthcare because they enhance accessibility, reliability, and cost effectiveness. Technology advancements that enable this expansion will include devices that acquire more reliable and higher quality signals and those that obtain more signals simultaneously, increasingly approximating clinical diagnostics. In terms of the wearable ECG, high-quality data will be continuously obtained from more reliable and improved sensors with multiple leads [1,5].

In the future, AI algorithms will be trained using an increasing number of larger, curated, condition-specific datasets. Future datasets that are more generalized to include more covariates to capture additional peripheral information are also likely to emerge. Data collected by wearable ECG devices will increasingly be transferred to a cloud for AI processing because the algorithms will be too computationally intensive to be executed locally [2,132].

Prospective wearable ECG-AI devices will normalize the near-instantaneous assessment and treatment of certain acute conditions, improving outcomes. These devices and algorithms will also more comprehensively consider whole-body physiology and health by integrating a variety of data sources simultaneously. Ongoing successes will increase confidence in automated decision making and reinforce its role in personalized healthcare [9,129].

## 8. Conclusions

The ECG contains highly valuable information. The diagnosing and predicting of specific clinical conditions, including arrhythmias, coronary artery disease, sleep apnea, mental health, and epilepsy are increasingly enabled via wearable devices that record ECG data and continuously analyze it in real-time using AI algorithms. In this review, we highlighted the current applications, with performances and limitations, of ECG-AI applied to wearable devices for disease detection and prediction. As reported by several other authors, the ongoing development of large, curated datasets targeting specific clinical conditions is essential for developing and validating various AI approaches. Since ECG-AI is tailored to specific medical applications, the methods that are most effective for one clinical condition are not necessarily appropriate for application to others. Advancements in this field require a combination of knowledge domains that create a unique expertise. Such technology is leading to a paradigm shift in personalized medicine that is making the diagnosis of many conditions more accessible, reliable, and cost effective.

## Data Availability

Not applicable.

## Abbreviations

Acc	accuracy
AF	atrial fibrillation
AI	artificial intelligence
CSA	central sleep apnea
CNN	convolutional neural network
CVD	cardiovascular diseases
DL	deep learning
DNN	deep learning neural network
ECG	electrocardiogram
ECG-AI	artificial-intelligence-enhanced electrocardiography
EEG	electroencephalogram
FDA	Food and Drug Administration
HF	heart failure
HR	heart rate
ML	machine learning
LSTM	long short-term Memory
OSA	obstructive sleep apnea
PPG	photoplethysmography
PVC	premature ventricular contraction
RNN	recurrent neural networks
SVM	support vector machine

## References

[1] Dagher L., Shi H., Zhao Y., Marrouche N.F. (2020). Wearables in cardiology: Here to stay. Heart Rhythm.

[2] Lu L., Zhang J., Xie Y., Gao F., Xu S., Wu X., Ye Z. (2020). Wearable Health Devices in Health Care: Narrative Systematic Review. JMIR mHealth uHealth.

[3] Duncker D., Ding W.Y., Etheridge S., Noseworthy P.A., Veltmann C., Yao X., Bunch T.J., Gupta D. (2021). Smart Wearables for Cardiac Monitoring-Real-World Use beyond Atrial Fibrillation. Sensors.

[4] Kumari P., Mathew L., Syal P. (2017). Increasing trend of wearables and multimodal interface for human activity monitoring: A review. Biosens. Bioelectron..

[5] Gargiulo G.D., Naik G.R. (2022). Wearable/Personal Monitoring Devices Present to Future.

[6] Witvliet M.P., Karregat E.P.M., Himmelreich J.C.L., de Jong J.S.S.G., Lucassen W.A.M., Harskamp R.E. (2021). Usefulness, pitfalls and interpretation of handheld single-lead electrocardiograms. J. Electrocardiol..

[7] Attia Z.I., Harmon D.M., Behr E.R., Friedman P.A. (2021). Application of artificial intelligence to the electrocardiogram. Eur. Heart J..

[8] Feeny A.K., Chung M.K., Madabhushi A., Attia Z.I., Cikes M., Firouznia M., Friedman P.A., Kalscheur M.M., Kapa S., Narayan S.M. (2020). Artificial Intelligence and Machine Learning in Arrhythmias and Cardiac Electrophysiology. Circ. Arrhythmia Electrophysiol..

[9] Hamet P., Tremblay J. (2017). Artificial intelligence in medicine. Metabolism.

[10] Koulaouzidis G., Jadczyk T., Iakovidis D.K., Koulaouzidis A., Bisnaire M., Charisopoulou D. (2022). Artificial Intelligence in Cardiology-A Narrative Review of Current Status. J. Clin. Med..

[11] (2022). Artificial Intelligence and Machine Learning (AI/ML)-Enabled Medical Devices. FDA. https://www.fda.gov/medical-devices/software-medical-device-samd/artificial-intelligence-and-machine-learning-aiml-enabled-medical-devices.

[12] Gibson C.M., Mehta S., Ceschim M.R., Frauenfelder A., Vieira D., Botelho R., Fernandez F., Villagran C., Niklitschek S., Matheus C.I. (2022). Evolution of single-lead ECG for STEMI detection using a deep learning approach. Int. J. Cardiol..

[13] Shao M., Zhou Z., Bin G., Bai Y., Wu S. (2020). A Wearable Electrocardiogram Telemonitoring System for Atrial Fibrillation Detection. Sensors.

[14] Fu Z., Hong S., Zhang R., Du S. (2021). Artificial-Intelligence-Enhanced Mobile System for Cardiovascular Health Management. Sensors.

[15] Cai W., Chen Y., Guo J., Han B., Shi Y., Ji L., Wang J., Zhang G., Luo J. (2020). Accurate detection of atrial fibrillation from 12-lead ECG using deep neural network. Comput. Biol. Med..

[16] Baloglu U.B., Talo M., Yildirim O., Tan R.S., Acharya U.R. (2019). Classification of myocardial infarction with multi-lead ECG signals and deep CNN. Pattern Recognit. Lett..

[17] Meng L., Tan W., Ma J., Wang R., Yin X., Zhang Y. (2022). Enhancing dynamic ECG heartbeat classification with lightweight transformer model. Artif. Intell. Med..

[18] Yu J., Wang X., Chen X., Guo J. (2021). Automatic Premature Ventricular Contraction Detection Using Deep Metric Learning and KNN. Biosensors.

[19] Ellenbogen K.A. (2021). Josephson’s Clinical Cardiac Electrophysiology. JACC Clin. Electrophysiol..

[20] Lip G.Y., Nieuwlaat R., Pisters R., Lane D.A., Crijns H.J. (2010). Refining Clinical Risk Stratification for Predicting Stroke and Thromboembolism in Atrial Fibrillation Using a Novel Risk Factor-Based Approach. Chest.

[21] Gopinathannair R., Sullivan R.M., Olshansky B. (2009). Tachycardia-mediated cardiomyopathy: Recognition and management. Curr. Heart Fail. Rep..

[22] Kornej J., Börschel C.S., Benjamin E.J., Schnabel R.B. (2020). Epidemiology of Atrial Fibrillation in the 21st Century. Circ. Res..

[23] Bashar S.K., Han D., Zieneddin F., Ding E., Fitzgibbons T.P., Walkey A.J., McManus D.D., Javidi B., Chon K.H. (2021). Novel Density Poincaré Plot Based Machine Learning Method to Detect Atrial Fibrillation from Premature Atrial/Ventricular Contractions. IEEE Trans. Biomed. Eng..

[24] Wang J. (2021). Automated detection of premature ventricular contraction based on the improved gated recurrent unit network. Comput. Methods Programs Biomed..

[25] Zipes D.P., Wellens H.J.J. (1998). Sudden Cardiac Death. Circulation.

[26] Malakar A.K., Choudhury D., Halder B., Paul P., Uddin A., Chakraborty S. (2019). A review on coronary artery disease, its risk factors, and therapeutics. J. Cell. Physiol..

[27] Tsao C.W., Aday A.W., Almarzooq Z.I., Alonso A., Beaton A.Z., Bittencourt M.S., Boehme A.K., Buxton A.E., Carson A.P., Commodore-Mensah Y. (2022). Heart Disease and Stroke Statistics—2022 Update: A Report from the American Heart Association. Circulation.

[28] Zipes M. (2019). Braunwald’s Heart Disease: A Textbook of Cardiovascular Medicine. https://evolve.elsevier.com/cs/product/9780323611886?role=student.

[29] Mannhart D., Lischer M., Knecht S., Lavallaz J.D.F.D., Strebel I., Serban T., Vögeli D., Schaer B., Osswald S., Mueller C. (2023). Clinical Validation of 5 Direct-to-Consumer Wearable Smart Devices to Detect Atrial Fibrillation. JACC Clin. Electrophysiol..

[30] Chen E., Jiang J., Su R., Gao M., Zhu S., Zhou J., Huo Y. (2020). A new smart wristband equipped with an artificial intelligence algorithm to detect atrial fibrillation. Heart Rhythm.

[31] Panganiban E.B., Paglinawan A.C., Chung W.Y., Paa G.L.S. (2021). ECG diagnostic support system (EDSS): A deep learning neural network based classification system for detecting ECG abnormal rhythms from a low-powered wearable biosensors. Sens. Bio-Sens. Res..

[32] Lown M., Brown M., Brown C., Yue A.M., Shah B.N., Corbett S.J., Lewith G., Stuart B., Moore M., Little P. (2020). Machine learning detection of Atrial Fibrillation using wearable technology. PLoS ONE.

[33] Khan M.A., Abbas S., Atta A., Ditta A., Alquhayz H., Rahman A.U., Naqvi R.A. (2020). Intelligent Cloud Based Heart Disease Prediction System Empowered with Supervised Machine Learning. Comput. Mater. Contin..

[34] Wasimuddin M., Elleithy K., Abuzneid A., Faezipour M., Abuzaghleh O. (2021). Multiclass ECG Signal Analysis Using Global Average-Based 2-D Convolutional Neural Network Modeling. Electronics.

[35] Chowdhury M.E.H., Alzoubi K., Khandakar A., Khallifa R., Abouhasera R., Koubaa S., Ahmed R., Hasan A. (2019). Wearable Real-Time Heart Attack Detection and Warning System to Reduce Road Accidents. Sensors.

[36] Perez M.V., Mahaffey K.W., Hedlin H., Rumsfeld J.S., Garcia A., Ferris T., Balasubramanian V., Russo A.M., Rajmane A., Cheung L. (2019). Large-Scale Assessment of a Smartwatch to Identify Atrial Fibrillation. N. Engl. J. Med..

[37] Fu W., Li R. (2021). Diagnostic performance of a wearing dynamic ECG recorder for atrial fibrillation screening: The HUAMI heart study. BMC Cardiovasc. Disord..

[38] Santala O.E., Halonen J., Martikainen S., Jäntti H., Rissanen T.T., Tarvainen M.P., Laitinen T.P., Laitinen T.M., Väliaho E.-S., Hartikainen J.E.K. (2021). Automatic Mobile Health Arrhythmia Monitoring for the Detection of Atrial Fibrillation: Prospective Feasibility, Accuracy, and User Experience Study. JMIR mHealth uHealth.

[39] Jeon E., Oh K., Kwon S., Son H., Yun Y., Jung E.-S., Kim M.S. (2020). A Lightweight Deep Learning Model for Fast Electrocardiographic Beats Classification with a Wearable Cardiac Monitor: Development and Validation Study. JMIR Public Health Surveill..

[40] Bazi Y., Al Rahhal M.M., AlHichri H., Ammour N., Alajlan N., Zuair M. (2020). Real-Time Mobile-Based Electrocardiogram System for Remote Monitoring of Patients with Cardiac Arrhythmias. Int. J. Pattern Recognit. Artif. Intell..

[41] Ma C., Wei S., Chen T., Zhong J., Liu Z., Liu C. (2021). Integration of Results from Convolutional Neural Network in a Support Vector Machine for the Detection of Atrial Fibrillation. IEEE Trans. Instrum. Meas..

[42] Goldberger A.L., Amaral L.A.N., Glass L., Hausdorff J.M., Ivanov P.C., Mark R.G., Mietus J.E., Moody G.B., Peng C.-K., Stanley H.E. (2000). PhysioBank, PhysioToolkit, and PhysioNet: Components of a New Research Resource for Complex Physiologic Signals. Circulation.

[43] Moody G.B., Mark R.G. (2001). The impact of the MIT-BIH Arrhythmia Database. IEEE Eng. Med. Biol. Mag..

[44] Lee K.-S., Park H.-J., Kim J.E., Kim H.J., Chon S., Kim S., Jang J., Kim J.-K., Jang S., Gil Y. (2022). Compressed Deep Learning to Classify Arrhythmia in an Embedded Wearable Device. Sensors.

[45] Wu X., Zheng Y., Chu C.-H., He Z. (2020). Extracting deep features from short ECG signals for early atrial fibrillation detection. Artif. Intell. Med..

[46] Ben Itzhak S., Ricon S.S., Biton S., Behar J.A., Sobel J.A. (2022). Effect of temporal resolution on the detection of cardiac arrhythmias using HRV features and machine learning. Physiol. Meas..

[47] Mei Z., Gu X., Chen H., Chen W. (2018). Automatic Atrial Fibrillation Detection Based on Heart Rate Variability and Spectral Features. IEEE Access.

[48] Smisek R., Hejc J., Ronzhina M., Nemcova A., Marsanova L., Kolarova J., Smital L., Vitek M. (2018). Multi-stage SVM approach for cardiac arrhythmias detection in short single-lead ECG recorded by a wearable device. Physiol. Meas..

[49] Tang X., Ma Z., Hu Q., Tang W. (2020). A Real-Time Arrhythmia Heartbeats Classification Algorithm Using Parallel Delta Modulations and Rotated Linear-Kernel Support Vector Machines. IEEE Trans. Biomed. Eng..

[50] Hua J., Zhang H., Liu J., Xu Y., Guo F. (2018). Direct Arrhythmia Classification from Compressive ECG Signals in Wearable Health Monitoring System. J. Circuits Syst. Comput..

[51] Pławiak P., Acharya U.R. (2020). Novel deep genetic ensemble of classifiers for arrhythmia detection using ECG signals. Neural Comput. Appl..

[52] Yıldırım Ö., Pławiak P., Tan R.-S., Acharya U.R. (2018). Arrhythmia detection using deep convolutional neural network with long duration ECG signals. Comput. Biol. Med..

[53] Karthiga S., Abirami A.M. (2022). Deep Learning Convolutional Neural Network for ECG Signal Classification Aggregated Using IoT. Comput. Syst. Sci. Eng..

[54] Zhang Y., Liu S., He Z., Zhang Y., Wang C. (2022). A CNN Model for Cardiac Arrhythmias Classification Based on Individual ECG Signals. Cardiovasc. Eng. Technol..

[55] Ramesh J., Solatidehkordi Z., Aburukba R., Sagahyroon A. (2021). Atrial Fibrillation Classification with Smart Wearables Using Short-Term Heart Rate Variability and Deep Convolutional Neural Networks. Sensors.

[56] Chen Y., Zhang C., Liu C., Wang Y., Wan X. (2022). Atrial Fibrillation Detection Using a Feedforward Neural Network. J. Med. Biol. Eng..

[57] Huang Y., Li H., Yu X. (2021). A multiview feature fusion model for heartbeat classification. Physiol. Meas..

[58] Shin S., Kang M., Zhang G., Jung J., Kim Y.T. (2022). Lightweight Ensemble Network for Detecting Heart Disease Using ECG Signals. Appl. Sci..

[59] Mazumder O., Banerjee R., Roy D., Mukherjee A., Ghose A., Khandelwal S., Sinha A. (2021). Computational Model for Therapy Optimization of Wearable Cardioverter Defibrillator: Shockable Rhythm Detection and Optimal Electrotherapy. Front. Physiol..

[60] Tan L., Yu K., Bashir A.K., Cheng X., Ming F., Zhao L., Zhou X. (2021). Toward real-time and efficient cardiovascular monitoring for COVID-19 patients by 5G-enabled wearable medical devices: A deep learning approach. Neural Comput. Appl..

[61] Li Y., Pang Y., Wang J., Li X. (2018). Patient-specific ECG classification by deeper CNN from generic to dedicated. Neurocomputing.

[62] Fan X., Yao Q., Cai Y., Miao F., Sun F., Li Y. (2018). Multiscaled Fusion of Deep Convolutional Neural Networks for Screening Atrial Fibrillation from Single Lead Short ECG Recordings. IEEE J. Biomed. Health Inform..

[63] Zhang P., Ma C., Sun Y., Fan G., Song F., Feng Y., Zhang G. (2021). Global hybrid multi-scale convolutional network for accurate and robust detection of atrial fibrillation using single-lead ECG recordings. Comput. Biol. Med..

[64] Sakib S., Fouda M.M., Fadlullah Z.M., Nasser N., Alasmary W. (2021). A Proof-of-Concept of Ultra-Edge Smart IoT Sensor: A Continuous and Lightweight Arrhythmia Monitoring Approach. IEEE Access.

[65] Ribeiro H.D.M., Arnold A., Howard J.P., Shun-Shin M.J., Zhang Y., Francis D.P., Lim P.B., Whinnett Z., Zolgharni M. (2022). ECG-based real-time arrhythmia monitoring using quantized deep neural networks: A feasibility study. Comput. Biol. Med..

[66] Ran S., Yang X., Liu M., Zhang Y., Cheng C., Zhu H., Yuan Y. (2022). Homecare-Oriented ECG Diagnosis with Large-Scale Deep Neural Network for Continuous Monitoring on Embedded Devices. IEEE Trans. Instrum. Meas..

[67] Qaisar S.M., Hussain S.F. (2020). Arrhythmia Diagnosis by Using Level-Crossing ECG Sampling and Sub-Bands Features Extraction for Mobile Healthcare. Sensors.

[68] Qaisar S.M., Subasi A. (2020). Cloud-based ECG monitoring using event-driven ECG acquisition and machine learning techniques. Phys. Eng. Sci. Med..

[69] Qaisar S.M., Mihoub A., Krichen M., Nisar H. (2021). Multirate Processing with Selective Subbands and Machine Learning for Efficient Arrhythmia Classification. Sensors.

[70] Cheng Y., Hu Y., Hou M., Pan T., He W., Ye Y. (2020). Atrial Fibrillation Detection Directly from Compressed ECG with the Prior of Measurement Matrix. Information.

[71] Zhang H., Dong Z., Gao J., Lu P., Wang Z. (2020). Automatic screening method for atrial fibrillation based on lossy compression of the electrocardiogram signal. Physiol. Meas..

[72] Alqudah A.M., Alqudah A. (2022). Deep learning for single-lead ECG beat arrhythmia-type detection using novel iris spectrogram representation. Soft Comput..

[73] Lee H., Shin M. (2021). Learning Explainable Time-Morphology Patterns for Automatic Arrhythmia Classification from Short Single-Lead ECGs. Sensors.

[74] Seo W., Kim N., Kim S., Lee C., Park S.-M. (2019). Deep ECG-Respiration Network (DeepER Net) for Recognizing Mental Stress. Sensors.

[75] Alqudah A.M., Qazan S., Al-Ebbini L., Alquran H., Abu Qasmieh I. (2022). ECG heartbeat arrhythmias classification: A comparison study between different types of spectrum representation and convolutional neural networks architectures. J. Ambient. Intell. Humaniz. Comput..

[76] Dami S., Yahaghizadeh M. (2021). Predicting cardiovascular events with deep learning approach in the context of the internet of things. Neural Comput. Appl..

[77] Khan M.A. (2020). An IoT Framework for Heart Disease Prediction Based on MDCNN Classifier. IEEE Access.

[78] Sopic D., Aminifar A., Atienza D. (2018). Real-Time Event-Driven Classification Technique for Early Detection and Prevention of Myocardial Infarction on Wearable Systems. IEEE Trans. Biomed. Circuits Syst..

[79] Shahnawaz M.B., Dawood H. (2021). An Effective Deep Learning Model for Automated Detection of Myocardial Infarction Based on Ultrashort-Term Heart Rate Variability Analysis. Math. Probl. Eng..

[80] Martin H., Morar U., Izquierdo W., Cabrerizo M., Cabrera A., Adjouadi M. (2021). Real-time frequency-independent single-Lead and single-beat myocardial infarction detection. Artif. Intell. Med..

[81] Cao Y., Wei T., Zhang B., Lin N., Rodrigues J.J.P.C., Li J., Zhang D. (2021). ML-Net: Multi-Channel Lightweight Network for Detecting Myocardial Infarction. IEEE J. Biomed. Health Inform..

[82] Cho J., Lee B., Kwon J.-M., Lee Y., Park H., Oh B.-H., Jeon K.-H., Park J., Kim K.-H. (2021). Artificial Intelligence Algorithm for Screening Heart Failure with Reduced Ejection Fraction Using Electrocardiography. ASAIO J..

[83] Cowie M.R. (2017). Sleep apnea: State of the art. Trends Cardiovasc. Med..

[84] Roberts E.G., Raphelson J.R., Orr J.E., LaBuzetta J.N., Malhotra A. (2022). The Pathogenesis of Central and Complex Sleep Apnea. Curr. Neurol. Neurosci. Rep..

[85] Pham L.V., Jun J., Polotsky V.Y., Chen R., Guyenet P.G. (2022). Chapter 6—Obstructive sleep apnea. Handbook of Clinical Neurology.

[86] Malhotra A., Ayappa I., Ayas N., Collop N., Kirsch D., Mcardle N., Mehra R., Pack A.I., Punjabi N., White D.P. (2021). Metrics of sleep apnea severity: Beyond the apnea-hypopnea index. Sleep.

[87] Benjafield A.V., Ayas N.T., Eastwood P.R., Heinzer R., Ip M.S.M., Morrell M.J., Nunez C.M., Patel S.R., Penzel T., Pépin J.-L. (2019). Estimation of the global prevalence and burden of obstructive sleep apnoea: A literature-based analysis. Lancet Respir. Med..

[88] de Zambotti M., Menghini L., Grandner M.A., Redline S., Zhang Y., Wallace M.L., Buxton O.M. (2022). Rigorous performance evaluation (previously, “validation”) for informed use of new technologies for sleep health measurement. Sleep Health.

[89] Penzel T., Moody G.B., Mark R.G., Goldberger A.L., Peter J.H. (2000). The apnea-ECG database. Comput. Cardiol..

[90] Wang T., Lu C., Shen G., Hong F. (2019). Sleep apnea detection from a single-lead ECG signal with automatic feature-extraction through a modified LeNet-5 convolutional neural network. PeerJ.

[91] Urtnasan E., Park J.-U., Joo E.Y., Lee K.J. (2020). Identification of Sleep Apnea Severity Based on Deep Learning from a Short-term Normal ECG. J. Korean Med. Sci..

[92] Shen Q., Qin H., Wei K., Liu G. (2021). Multiscale Deep Neural Network for Obstructive Sleep Apnea Detection Using RR Interval From Single-Lead ECG Signal. IEEE Trans. Instrum. Meas..

[93] Almutairi H., Hassan G.M., Datta A. (2021). Classification of Obstructive Sleep Apnoea from single-lead ECG signals using convolutional neural and Long Short Term Memory networks. Biomed. Signal Process. Control.

[94] Bahrami M., Forouzanfar M. (2022). Deep Learning Forecasts the Occurrence of Sleep Apnea from Single-Lead ECG. Cardiovasc. Eng. Technol..

[95] Qin H., Liu G. (2022). A dual-model deep learning method for sleep apnea detection based on representation learning and temporal dependence. Neurocomputing.

[96] Yang Q., Zou L., Wei K., Liu G. (2021). Obstructive sleep apnea detection from single-lead electrocardiogram signals using one-dimensional squeeze-and-excitation residual group network. Comput. Biol. Med..

[97] Cacioppo J.T., Tassinary L.G., Berntson G.G., Wager T.D., Hernandez L., Jonides J., Lindquist M., Pizzagalli D.A., Fabiani M., Gratton G., Cacioppo J.T., Tassinary L.G., Berntson G.G. (2007). Psychophysiological Science: Interdisciplinary Approaches to Classic Questions about the Mind. The Handbook of Psychophysiology.

[98] O’Connor D.B., Thayer J.F., Vedhara K. (2021). Stress and Health: A Review of Psychobiological Processes. Annu. Rev. Psychol..

[99] Krantz D.S., Manuck S.B. (1984). Acute psychophysiologic reactivity and risk of cardiovascular disease: A review and methodologic critique. Psychol. Bull..

[100] Schwartz A.R., Gerin W., Davidson K., Pickering T.G., Brosschot J.F., Thayer J.F., Christenfeld N., Linden W. (2003). Toward a Causal Model of Cardiovascular Responses to Stress and the Development of Cardiovascular Disease. Psychosom. Med..

[101] Dedovic K., Ngiam J. (2015). The cortisol awakening response and major depression: Examining the evidence. Neuropsychiatr. Dis. Treat..

[102] Chida Y., Hamer M. (2008). Chronic psychosocial factors and acute physiological responses to laboratory-induced stress in healthy populations: A quantitative review of 30 years of investigations. Psychol. Bull..

[103] Chauvet-Gelinier J.-C., Bonin B. (2017). Stress, anxiety and depression in heart disease patients: A major challenge for cardiac rehabilitation. Ann. Phys. Rehabil. Med..

[104] Castaldo R., Melillo P., Bracale U., Caserta M., Triassi M., Pecchia L. (2015). Acute mental stress assessment via short term HRV analysis in healthy adults: A systematic review with meta-analysis. Biomed. Signal Process. Control.

[105] Parlak O. (2021). Portable and wearable real-time stress monitoring: A critical review. Sens. Actuators Rep..

[106] Bali A., Jaggi A.S. (2015). Clinical experimental stress studies: Methods and assessment. Rev. Neurosci..

[107] Shiban Y., Diemer J., Brandl S., Zack R., Mühlberger A., Wüst S. (2016). Trier Social Stress Test in vivo and in virtual reality: Dissociation of response domains. Int. J. Psychophysiol..

[108] Wilhelm F.H., Grossman P. (2010). Emotions beyond the laboratory: Theoretical fundaments, study design, and analytic strategies for advanced ambulatory assessment. Biol. Psychol..

[109] Shaffer F., McCraty R., Zerr C.L. (2014). A healthy heart is not a metronome: An integrative review of the heart’s anatomy and heart rate variability. Front. Psychol..

[110] Thayer J.F., Hansen A.L., Saus-Rose E., Johnsen B.H. (2009). Heart Rate Variability, Prefrontal Neural Function, and Cognitive Performance: The Neurovisceral Integration Perspective on Self-regulation, Adaptation, and Health. Ann. Behav. Med..

[111] Berntson G.G., Bigger J.T., Eckberg D.L., Grossman P., Kaufmann P.G., Malik M., Nagaraja H.N., Porges S.W., Saul J.P., Stone P.H. (1997). Heart rate variability: Origins, methods, and interpretive caveats. Psychophysiology.

[112] Schulze-Bonhage A., Sales F., Wagner K., Teotonio R., Carius A., Schelle A., Ihle M. (2010). Views of patients with epilepsy on seizure prediction devices. Epilepsy Behav..

[113] Lotufo P.A., Valiengo L., Benseñor I.M., Brunoni A.R. (2012). A systematic review and meta-analysis of heart rate variability in epilepsy and antiepileptic drugs. Epilepsia.

[114] Eggleston K.S., Olin B.D., Fisher R.S. (2014). Ictal tachycardia: The head–heart connection. Seizure.

[115] Betti S., Lova R.M., Rovini E., Acerbi G., Santarelli L., Cabiati M., Del Ry S., Cavallo F. (2018). Evaluation of an Integrated System of Wearable Physiological Sensors for Stress Monitoring in Working Environments by Using Biological Markers. IEEE Trans. Biomed. Eng..

[116] Cho H.-M., Park H., Dong S.-Y., Youn I. (2019). Ambulatory and Laboratory Stress Detection Based on Raw Electrocardiogram Signals Using a Convolutional Neural Network. Sensors.

[117] Huang S., Li J., Zhang P., Zhang W. (2018). Detection of mental fatigue state with wearable ECG devices. Int. J. Med. Inform..

[118] Healey J., Picard R. (2005). Detecting Stress During Real-World Driving Tasks Using Physiological Sensors. IEEE Trans. Intell. Transp. Syst..

[119] Sepúlveda A., Castillo F., Palma C., Rodriguez-Fernandez M. (2021). Emotion Recognition from ECG Signals Using Wavelet Scattering and Machine Learning. Appl. Sci..

[120] Yamakawa T., Miyajima M., Fujiwara K., Kano M., Suzuki Y., Watanabe Y., Watanabe S., Hoshida T., Inaji M., Maehara T. (2020). Wearable Epileptic Seizure Prediction System with Machine-Learning-Based Anomaly Detection of Heart Rate Variability. Sensors.

[121] Vandecasteele K., De Cooman T., Chatzichristos C., Cleeren E., Swinnen L., Ortiz J.M., Van Huffel S., Dümpelmann M., Schulze-Bonhage A., De Vos M. (2021). The power of ECG in multimodal patient-specific seizure monitoring: Added value to an EEG-based detector using limited channels. Epilepsia.

[122] Ihle M., Feldwisch-Drentrup H., Teixeira C., Witon A., Schelter B., Timmer J., Schulze-Bonhage A. (2012). EPILEPSIAE—A European epilepsy database. Comput. Methods Programs Biomed..

[123] Beniczky S., Ryvlin P. (2018). Standards for testing and clinical validation of seizure detection devices. Epilepsia.

[124] Malik M., Bigger J.T., Camm A.J., Kleiger R.E., Malliani A., Moss A.J., Schwartz P.J. (1996). Heart rate variability: Standards of measurement, physiological interpretation, and clinical use. Eur. Heart J..

[125] Porumb M., Stranges S., Pescapè A., Pecchia L. (2020). Precision Medicine and Artificial Intelligence: A Pilot Study on Deep Learning for Hypoglycemic Events Detection based on ECG. Sci. Rep..

[126] Cordeiro R., Karimian N., Park Y. (2021). Hyperglycemia Identification Using ECG in Deep Learning Era. Sensors.

[127] Luo X. (2021). ECG signal analysis for fatigue and abnormal event detection during sport and exercise. Internet Technol. Lett..

[128] Rajkomar A., Dean J., Kohane I. (2019). Machine Learning in Medicine. N. Engl. J. Med..

[129] Meskó B., Görög M. (2020). A short guide for medical professionals in the era of artificial intelligence. npj Digit. Med..

[130] Neri L., Oberdier M.T., Augello A., Suzuki M., Tumarkin E., Jaipalli S., Geminiani G.A., Halperin H.R., Borghi C. (2023). Algorithm for Mobile Platform-Based Real-Time QRS Detection. Sensors.

[131] Hossain B., Bashar S.K., Walkey A.J., McManus D.D., Chon K.H. (2019). An Accurate QRS Complex and P Wave Detection in ECG Signals Using Complete Ensemble Empirical Mode Decomposition with Adaptive Noise Approach. IEEE Access.

[132] Rajpurkar P., Chen E., Banerjee O., Topol E.J. (2022). AI in health and medicine. Nat. Med..

